# Effect of the Histone Methyltransferase Specific Probe BRD4770 on Metabolic Profiling of the Endophytic Fungus *Diaporthe longicolla*

**DOI:** 10.3389/fmicb.2021.725463

**Published:** 2021-09-17

**Authors:** Jay Hind Nishad, Arti Singh, Rajnish Bharti, Priyanka Prajapati, Vijay Kumar Sharma, Vijai Kumar Gupta, Ravindra Nath Kharwar

**Affiliations:** ^1^Mycopathology and Microbial Technology Laboratory, Centre of Advanced Study in Botany, Institute of Science, Banaras Hindu University, Varanasi, India; ^2^Agricultural Research Organization, Rishon LeZion, Israel; ^3^Center for Safe and Improved Food, Biorefining and Advanced Materials Research Center, Scotland’s Rural College, Edinburgh, United Kingdom

**Keywords:** endophytic fungus, epigenetic modulator, GC-MS, LC-ESI-MS/MS, reverse-phase HPLC

## Abstract

The endophytic fungus *Diaporthe longicolla* was isolated from the stem of *Saraca asoca* (Roxb.) Willd., commonly known as Ashok plant in India and Sri Lanka. Since no reports are available regarding epigenetic modulations by BRD4770 in microbial entities, *D*. *longicolla* was treated with different concentrations of BRD4770 for this purpose and evaluated for its antioxidant and antibacterial potential against five human pathogenic bacteria, *Staphylococcus aureus*, methicillin-resistant *Staphylococcus aureus* (MRSA), *Shigella boydii, Klebsiella pneumoniae*, and *Escherichia coli.* The crude extract obtained from cultures treated with 100 nM concentration of BRD4770 showed increased antioxidant activity and inhibition zone against *S. aureus* and MRSA, compared to the non-treated control. The composition of the non-treated and treated crude extract was analyzed, and induced compounds were identified with the help of Gas chromatography–mass spectrometry (GC-MS) and LC-ESI-MS/MS. LC-ESI-MS/MS analysis showed that berberine (antibacterial)-, caffeine-, and theobromine (antioxidant)-like compounds were induced in the BRD4770-treated crude extract. The presence of particular absorbance at a wavelength of 346.5 nm for berberine, 259.4 nm for caffeine, and 278.4 nm for theobromine in the reverse-phase high-performance liquid chromatography (HPLC) analysis of both BRD4770-treated crude metabolites and standard solution of the above compounds strongly supported the increased antibacterial and antioxidant activities that may be due to inducing the alterations in bioactivities of the BRD4770-treated culture.

## Highlights

- An endophytic fungus (L3), isolated from the leaf tissues of *Saraca asoca* was identified as *Diaporthe longicolla*.
- The crude metabolite extracts of *D. longicolla* showed strong antioxidant and antibacterial properties.
- *D. longicolla* was treated with different concentrations of BRD4770 for evaluation of its antibacterial and antioxidant potential.
- The crude extract obtained from cultures treated with 100 nM concentration of BRD4770, showed increased inhibition zone against *S. aureus* and methicillin-resistant *Staphylococcus aureus* (MRSA), compared to the non-treated control.
- Antioxidant activity was also founded to increase in treated with 100 nM BRD4770 compared to the non-treated culture.
- The composition of non-treated as well as treated crude extract was analyzed and induced compounds were identified with the help of GC-MS and LC-ESI-MS/MS.
- LC-ESI-MS/MS analysis showed berberine (antibacterial), caffeine, and theobromine (antioxidant) like compounds, etc., were induced in BRD4770 treated crude extract.
- Presence of particular absorbance at wavelength 346.5 for berberine, 259.4 for caffeine and 278.4 nm for theobromine in the reverse phase HPLC analysis of both BRD4770 treated crude metabolites and standard solution confirms its presence.

## Introduction

German scientist de Bary (1866) gave the term “endophytes” for any organism (bacteria, fungi, actinomycetes, etc.) that resides in healthy plant tissues internally, without causing any apparent disease symptoms to the host. Endophytes as an alternative source of plant-derived drugs like camptothecin, vincristine, vinblastine, rohitukine, azadirachtin, and piperine will reduce not only the price and crisis of host mimetic compounds but also the overexploitation of host plants (Kharwar et al., 2008, 2011, 2020; Verma et al., 2009, 2014; Singh et al., 2021). *Diaporthe longicolla*, a member of Ascomycota, belongs to class Dothideomycetes, order Diaporthales, and family Diaporthaceae (Bubák and Kabát, 1905). Various numbers of structurally diverse polyketides have also been reported from *D. longicolla*. These include cytoskyrins and mycotoxins that lead to DNA damage and inhibit the growth in *Escherichia coli* (Agusta et al., 2006). *D. longicolla* isolated from *Annona squamosa* produces compounds like diaporthemins A and B and flavomannin-6,6’-dimethylether that are antibacterial against *Streptococcus pneumoniae* (Ola et al., 2014). It has always been an interest of scientists to maximize the numbers and yield of compounds and also to receive cryptic metabolites from microbes by adopting different approaches such as substrate selection, co-culture, one strain many compounds (OSMAC), and epigenetic modulations.

In fungi, the biosynthetic potential of genes for secondary metabolites is typically organized in gene clusters, of which several clusters recognized for the biosynthesis of secondary metabolites remain silent (Rutledge and Challis, 2015). These silent or cryptic genes can be induced through epigenetic modulations to discover novel compounds. Epigenetic modulations can be used as a tool to stimulate an endophyte to produce more compounds with far greater bioactivities (Sharma et al., 2017). Treatment of fungi with histone deacetylase (HDAC) inhibitors like suberoylanilide hydroxamic acid (SAHA), sodium butyrate, and valproic acid, and DNA methyltransferase (DNMT) inhibitors like 5-aza-20-deoxycytidine 5-azacytidine, procainamide, procaine, and hydralazine has been shown to facilitate the induction of cryptic gene or activation of biosynthetic pathways of secondary metabolites. Similarly, methyl ester BRD4770 can significantly reduce cellular levels of H3K9me2 and H3K9me3, but may increase the cellular levels of H3K9me1 and activation of various cellular responses. These bioactive chemical probes alter the DNA methylation and histone modifications requisite for gene activation in the same way as the other chemical epigenetic modifiers (Meeran et al., 2010). Epigenetic influences of many chemical modulators have been much studied for the induction and activation of many known and new metabolites, but the effect of BRD4770 on the induction of secondary metabolites in fungi is still not known well. The BRD4770 is a novel histone methyltransferase inhibitor that can be instrumental in the epigenetic manipulation of silent gene clusters in fungal endophytes. This study aimed to use a histone methyltransferase (G9a), a specific probe BRD4770 either to enhance the activity of compounds or to isolate the cryptic metabolites from an endophytic fungus *D. longicolla* for the first time. The plant selected to isolate *D. longicolla* was *Saraca asoca* (Roxb.) Willd., a legendary and sacred tree of India, having one of the most fascinating flowers in the Indian range of flower essences.

## Materials and Methods

### Isolation, Identification, and Molecular Characterization of Endophytic Fungus

Isolation and identification of endophytic fungus *D. longicolla* were done from the mature healthy leaves of the *S. asoca* plant growing in the Botanical Garden of BHU (Banaras Hindu University) campus, Varanasi, India. Mature, green, asymptomatic leaves were randomly collected from the lowermost branches of the plant sealed in sterile polybags and kept in an icebox (4°C) until further processing. The modified protocol of Arnold and Lutzoni (2007) was adopted for surface sterilization (Petrini et al., 1992). Briefly, leaf was cut into 5 mm × 5 mm long segments and consecutively dipped in 70% ethanol for 1 min followed by 30 s in 4% sodium hypochlorite and finally rinsed thrice with autoclaved distilled water to remove excess surface sterilant. Under aseptic conditions, the tissue segments were dried before plating; four leaf segments were placed evenly onto PDA plates supplemented with streptomycin (50 μg/ml) to inhibit the growth of bacterial endophytes. Plates were sealed with parafilm and incubated in BOD incubator at 28°C ± 2°C for 5–7 days. Emerging fungal colonies were aseptically transferred to fresh PDA plates to obtain pure cultures. Primary identification of isolated endophytic fungus was done through morphology and reproductive structures observed under the microscope. Genotypic identification was done through total genomic DNA extraction (DNA kit, GeNei) from fresh mycelium cultured in potato dextrose broth (PDB). Polymerase chain reaction (PCR) was performed using extracted DNA with universal primers ITS1: TCCGT AGGTGAACCTGCGG and ITS4: TCCTCCGCTTGATATGC. Total PCR mixture for 25 μl, containing 1 μl of each primer, 0.33 μl (3 units/μl) of Taq polymerase, 0.5 μl of dNTPs, 2.5 μl of 10 × PCR buffer with 25 mM MgCl_2_, 1 μl (100 ng/ml) of DNA template, and 18.67 μl of milli-Q water. PCR was performed using MyCycler (Bio-Rad, Hercules, CA, United States), with pre-denaturation at 94°C for 4 min; 35 cycles of each denaturation was at 94°C for 1 min, and annealing was at 55°C for 1 min while extension was at 72°C for 1 min; final extension was at 72°C for 5 min. Amplified products were resolved by 1.5% (w/v) agarose using electrophoresis and gel was stained with ethidium bromide (0.5 mg/ml) for visual examination. PCR amplified DNA was further purified through gel excision method from Real Biotech Corporation (RBC, India) using a HiYield PCR DNA mini kit and sequenced by EDZ Daignon Pvt., Ltd., India. The obtained ITS rRNA gene sequences were used to retrieve similar sequences using the NCBI nBLAST program from the NCBI GenBank sequence database. The rRNA gene sequence was submitted to the NCBI GenBank database for accession number.

### Preparation of BRD4770 Solution for Epigenetic Treatment

Stock solution (100 μM) was prepared by dissolving 4.13 mg of BRD4770 in 100 ml DMSO and filtered with 0.20 mm filter paper for removal of any contaminant. PDB medium (100 ml) was amended with BRD4770 separately in a series of final concentrations of 1, 10, 25, 50, 100, 200, 400, and 1,000 nM, respectively. Finally, each test culture was inoculated with the endophytic fungus and incubated at 26°C ± 2°C for 21 days. The treatments were done in triplicate for reproducibility.

### Isolation of Secondary Metabolites

The selected culture of endophytic fungus was cultured in a 250-ml Erlenmeyer flask containing 100 ml of PDB medium for 21 days at 28°C and 150 rpm. The fermentation broth culture was filtered under clean and aseptic conditions. Extraction of the secondary metabolite from the filtrate was carried out thrice with the same volume of ethyl acetate as organic solvent. The solvent was added to the filtrate with 1:1 (v/v) ratio in a separating funnel, mixed properly for 10–15 min and left for 5–6 min until the formation of two clear immiscible layers. The metabolites containing an upper layer of solvent was collected and evaporated using a rotator vacuum evaporator (IKA, Germany) to yield the dried crude metabolites. The secondary metabolites obtained from different treatments were measured and dissolved in methanol to the final concentration of 10 mg/ml.

### Chemicals and Materials

BRD4770 (Methyl-2-(benzoylamino)-1-(3-phenylpropyl)-1H-benzimidazole-5-carboxylate) (>98%) for the epigenetic modulation, caffeine (>98%), theobromine (>98%), and berberine (>98%) standards were purchased from Sigma-Aldrich Co. (St. Louis, MO, United States) for comparative analysis. Acetonitrile, methanol, 0.1% trifluoroacetic acid (TFA), and water (HPLC grade) were purchased from Merck Pvt., Ltd. (Mumbai, India). All the chemicals including solvents were of analytical grade.

### Preparation of Reagents and Standards

For the mobile phase, 60 parts of 0.1% TFA and 40 parts of acetonitrile were mixed to get 0.1% TFA:acetonitrile (60:40 v/v) as a final solution. The mixed mobile phase was then filtered through nylon membrane vacuum filtration (0.22 μm) and degassed by sonication. Samples were analyzed on a detector at wavelength of 346.5 nm for berberine, 259.4 nm for caffeine, and 264.1 nm for theobromine and an injection volume of 10 μl using RP-C18 column (Waters, 4.6 mm × 100 mm, 3.5 μm) with a run time of 20 min.

### Preparation of Standard Solutions

A standard stock solution was prepared by dissolving 100 mg of berberine, caffeine, and theobromine in a 100-ml volumetric flask containing 60 ml mobile phase and then sonicated for about 10 min and made up to 100 ml with mobile phase to get the primary standard stock solution containing 1 mg/ml of berberine, caffeine, and theobromine. Working standard solutions were prepared by further dilution with the mobile phase.

### Detection of Berberine, Caffeine, and Theobromine in the Extract of *D. longicolla* by RP-HPLC

High-performance liquid chromatography analysis was performed for the treated crude extract and the non-treated crude extract of *D. longicolla*, using RP-C18 column (Waters, 4.6 mm × 100 mm, 3.5 μm, made in Netherlands). Pre-run sample stabilization was done for about 20 min with mobile phase consisting of 0.1% TFA:acetonitrile (60:40 v/v). The flow rate was maintained at 1.0 ml/min and the injected volume was 20 μl. The peak detection was done at 346.5 nm for berberine, 259.4 nm for caffeine, and 264.1 nm for theobromine, respectively, and the retention time (RT) was recorded duly maintaining the ambient experimental conditions. The presence of caffeine, theobromine, and berberine chromatogram in the BRD4770-treated sample was matched with the chromatogram of standards solution. The identification of each compound was based on a combination of RT, absorption maxima at a given wavelength, and chromatogram matching.

### Antibacterial Activity

The metabolites extracted from all the different treatments were screened for the antibacterial activity against five human bacterial pathogens, methicillin-resistant *Staphylococcus aureus* (MRSA) IMS/GN11, *Shigella boydii* IMS/GN17, *S. aureus* ATCC 25923, *Klebsiella pneumoniae*, and *E. coli* IMS/GN19 using the disk diffusion method. The crude extract was loaded aseptically 100 μl (1 mg) to the sterilized filter paper disks and air-dried. A lawn of test bacterial culture was spread with a cotton swab onto the surface of solidified Mueller-Hinton agar (MHA) Petri plates. Sterile paper disks loaded with 1 mg of crude extract were placed over the lawn of bacterial culture on MHA plates. Positive control included similar paper disks impregnated with 10 μl of methanol and dried. Plates were incubated for 24 h at 35°C ± 2°C and then antibacterial activity including the minimum zone of inhibition was observed. Each test was performed in triplicate and the mean diameter of the minimum zones of inhibition with standard deviation was presented in the results (Table 1).

**TABLE 1:** Results of one-way ANOVA and Tukey’s HSD mean comparison examining the zone of inhibition against different bacteria using metabolite extracted from treated and non-treated fungus *D. longicolla.*

	**Mean zone of inhibition (mm) shown by crude metabolites of different cultures**								
	
**Bacterial species**	**Control**	**10 nm**	**25 nm**	**50 nm**	**100 nm**	**200 nm**	**1 μ m**	***F* statistics**	Tukey’s HSD mean comparison*
MRSA	7.66 ± 0.57	7.00 ± 1.00	6.00 ± 1.00	6.33 ± 0.57	12.33 ± 1.5	7.33 ± 1.52	8.66 ± 1.52	9.954	100 nm > 1 μm > 200 nm > control, 10 nm, 50 nm, 25 nm
*S. aureus*	7.66 ± 1.50	6.33 ± 0.57	6.33 ± 0.57	7.33 ± 0.57	11.33 ± 0.57	8.66 ± 0.57	9.33 ± 0.57	15.59	100 nm > control, 10 nm, 25 nm, 50 nm, 200 nm, 1 μm
*E. coli*	NA	NA	NA	NA	NA	NA	NA	NA	NA
*S. boydii*	NA	NA	NA	NA	NA	NA	NA	NA	NA
*K. pneumoniae*	NA	NA	NA	NA	NA	NA	NA	NA	NA

*Significant at α = 0.05, NA, not applicable.

### Antioxidant Activity

The antioxidant activity of treated as well as non-treated crude extract was determined using ABTS^+^ [2,2′-azino-bis(3-ethylbenzothiazoline-6-sulfonic acid)] radical scavenging method (Miller and Rice-Evans, 1997). Three milliliters of ABTS^+^ was allowed to react with various concentrations (12.5, 25, 50, 100, 200, and 400 μg/3 ml or 4.16, 8.33, 16.66, 33.33, 66.66, and 133.33/ml) (Figures 2A,B and Table 2) of treated and non-treated crude extract for 30 min, and absorbance was recorded at 734 nm. The O.D. of pure ABTS^+^ was used as control and percent inhibition was calculated using the following formula:


Inhibition⁢of⁢ABTS+%=ControlA-SampleA⁢orReference/ControlA×100

**FIGURE 1:**
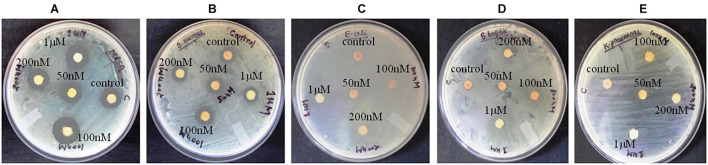
Antibacterial activity of crude compounds isolated from various concentration of BRD4770-treated and control (non-treated) cultures of *D. longicolla.* **(A)** Methicillin-resistant *Staphylococcus aureus.* **(B)** *Staphylococcus aureus.* **(C)** *Escherichia coli.* **(D)** *Shigella boydii.* **(E)** *Klebsiella pneumoniae*.

**FIGURE 2:**
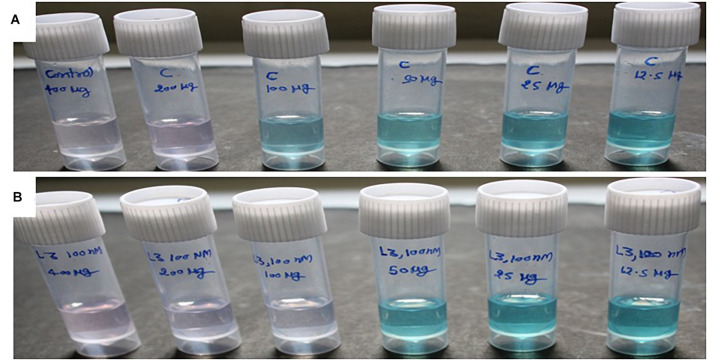
The ABTS^+^ radical scavenging method-based antioxidant activity of crude compounds in increasing concentration (μg/ml) isolated from *D. longicolla* **(A)** non-treated control and **(B)** BRD4770-treated cultures.

**TABLE 2:** ABTS^+^% inhibition of 100 nM BRD4770-treated and non-treated (control) crude metabolite.

**Crude metabolite concentration (μ g/ml)**	**ABTS^+^% inhibition**	
	**Control**	**BRD4770 treated**
4.16	1.51 ± 0.03	2.72 ± 0.40
8.33	3.24 ± 0.16	6.76 ± 0.68
16.66	7.47 ± 0.29	10.40 ± 0.57
33.33	13.63 ± 0.83	22.78 ± 0.74
66.66	26.35 ± 0.54	50.75 ± 0.75
133.33	47.27 ± 0.80	94.44 ± 0.48

*All % inhibition values are significant at α = 0.05.*

Where *^*A*^*Control and *^*A*^*Sample or Reference are the absorbance of the ABTS^+^ in the control solution and sample or reference solution, respectively. The antioxidant capacities were calculated based on calibration curves plotted using different concentrations of the sample and reference solution. All tests were performed in triplicate.

### Gas Chromatography–Mass Spectrometry Analysis Condition

Gas chromatography–mass spectrometry (GC-MS) analysis of the methanolic crude extracts was carried out in a (30 m 1D-0.25 mm film thickness-0.25 μm) capillary column of TSQ Duo Thermo Scientific a TG-5MS in BHU, Varanasi, India. The initial temperature of the instrument was set to 70°C and maintained for 3 min at this temperature. At the rate of an increase of 8°C/min, oven temperature was raised to 300°C at the end of this period, and maintained for 1 min. Helium flow rate was at 1 ml/min, and port of injection temperature was ensured at 250°C. The voltage of ionization was 70 eV. The samples were injected in a 40:1 split mode. Mass spectral scan range was set at 40–450 (m/z). The identification of bioactive compounds present in the crude extracts was performed by comparing the mass spectra with data from NIST05 (National Institute of Standards and Technology, United States) libraries. The name, molecular weight, and structure of the components of the test material were ascertained.

### HPLC-ESI-MS/MS Analysis Condition

The HPLC-ESI-MS/MS of the extracted crude was done on a MS system using a high-performance liquid chromatography (HPLC) system (Waters model no. ACQ-TQD#QBB 1152) fitted with a C18 column having dimensions of 100 × 2.1, 1.7 μm. The mobile phase flow ramp rate was 0.45 ml/min and the injection volume of the sample was 10 μl. The mobile phase was acetonitrile/water (5:95, v/v), 5 mM ammonium acetate of pH 6.5 buffer, and methanol solutions. Run time was 30 min, resolution 1.2 nm, range 210–800 nm. MS scan was started at 150 mass and ended at 2000 mass under + ESI source and MS-MS collision energy was 2.0. HPLC eluted peaks were recorded at different RTs. The fractions were finally characterized by mass spectrometry. This analysis was carried out at Sophisticated Analytical Instrumentation Facility (SAIF), CSIR-Central Drug Research Institute (CDRI), Lucknow, India. The identification of bioactive compounds was performed by comparing the mass spectra obtained at a particular RT with mass spectra of standard compounds stored in the spectral library of mzCloud spectral Database.

## Results

Based on microscopic as well as gene sequencing of ITS rRNA data, the isolate L3 was identified as *D. longicolla* with GenBank accession number MN480529.

### Antibacterial Activity of Treated and Non-treated Culture

Antibacterial activity of crude extract obtained from the 100 nM BRD4770-treated culture showed significantly higher inhibition zone (11.33 ± 0.57 and 12.33 ± 1.5) against *S. aureus* and MRSA, respectively, as compared to the non-treated crude extract (Figure 1 and Table 1). No zone of inhibition was observed against the rest of the other bacterial pathogens used for the assay, neither in control nor in the treated ones (Table 1).

### Antioxidant Activity of Crude Metabolite

ABTS^+^ radical scavenging activity was observed as% inhibition in crude metabolites of the control and treated culture (Figure 2 and Table 2). The calculated EC_50_ for the control was >133.33 μg/ml and that for the 100 nM BRD4770-treated culture was 66.66 μg/ml.

### Characterization of Bioactive Compounds

**FIGURE 3:**
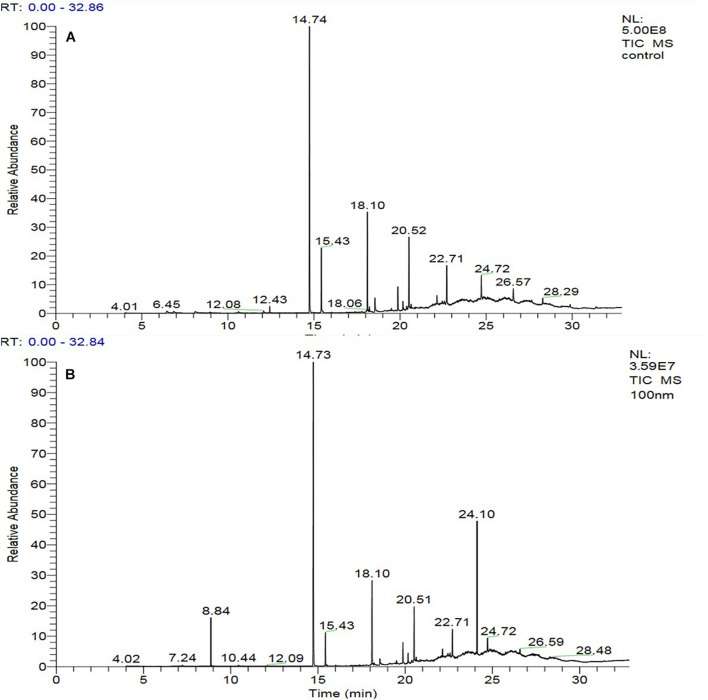
GC-MS chromatogram of crude metabolites of *D. longicolla* cultures **(A)** non-treated and **(B)** 100 nM BRD4770 treated.

The GC-MS of the crude ethyl acetate extracts of the 100 nM BRD4770-treated cultures was found to induce in secondary metabolites biosynthesis, viz., benzoic acid, 4-methyl-2-trimethylsilyloxy, and (4-chloro-3-nitrophenyl)methanol, dimethylpentafluorophenylsilyl ether (Figure 3B), while crude secondary metabolites from the rest of the treatments including non-treated ones were approximately similar (Supplementary Figure 2) in composition (Figure 3A). Details of the compounds identified by GC-MS analysis in the (100 nM) treated and non-treated culture are provided in Tables 3, 4.

**TABLE 3:** Induced compound in *D. longicolla* culture treated with 100 nM BRD4770.

**RT**	**Induced compound**	**Molecular formula**	**M.W.**	**Activity**	**References**
8.84	Benzoic acid, 4-methyl-2-trimethylsilyloxy	C_14_H_24_O_3_Si_2_	296.51	Antimicrobial	Foo et al., 2017
24.10	(4-Chloro-3-nitrophenyl)methanol, dimethylpentafluorophenylsilyl ether	C_15_H_11_ClF_5_NO_3_Si	411.783	–	–

*–, not available in literature.*

**TABLE 4:** Common compounds that were found in the fungal crude extract after treatment.

**RT**	**Compound name**	**Molecular formula**	**M.W**	**Activity**	**References**
14.73	2,4-Di-tert-butylphenol	C_14_H_22_O	206.32	Antioxidant, antibacterial	Viszwapriya et al., 2016
15.43	1-Hexadecanol	C_16_H_34_O	242.44	–	–
18.10	Acetic acid, 2-phenylethyl ester	C_10_H_12_O**_2_**	164.2	Antibacterial	Al-Dhabi et al., 2019
20.52	Heptacosene	C_27_H_54_	378.729	Antibacterial	Naeim et al., 2020
22.71	1-Hexadecanol, 2-methyl	C_17_H_36_O	256.5	–	–
24.72	17-Pentatriacontene	C_35_H_70_	490.9	Antioxidant	Sisin et al., 2017
26.57	Oleic acid, eicosyl ester	C_38_H_74_O_2_	562.99	–	–

### Identification of Compounds by HPLC-ESI-MS/MS

In full scan mass spectra of fungal metabolite, [M-H]^–^ was observed in negative ion ESI mode and [M+H]^+^ was observed in the positive ion ESI mode. The information on [M-H]^–^ and [M+H]^+^ was used to determine molecular weight.

**FIGURE 4:**
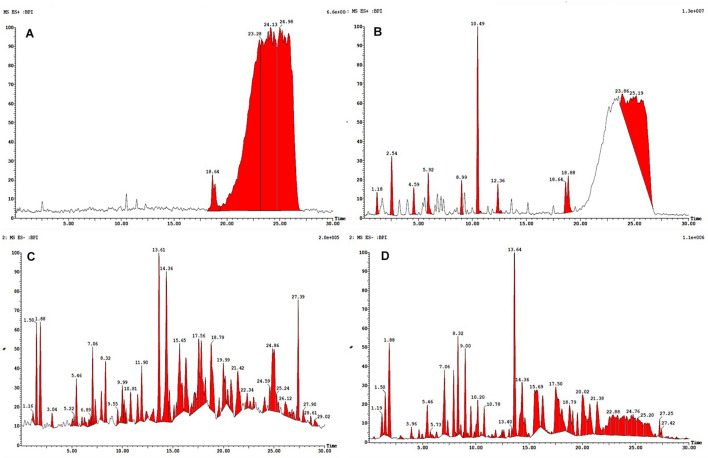
HPLC-ESI-MS/MS base peak chromatogram (BPC) of metabolite **(A)** non-treated (positive), **(B)** treated with 100 nM BRD4770 (positive), **(C)** non-treated (negative), and **(D)** treated with 100 nM BRD4770 (negative).

All the compounds were identified by the interpretation of their mass spectral behavior obtained from HPLC-ESI-MS/MS spectra, the accurate mass, and observed MS*^*n*^* fragments that were acquired from ESI^+^ and ESI^–^ mode and also taking into account data provided by the literature. Raw data of HPLC-ESI-MS/MS were analyzed with ACD/Spectrus software to obtain the top 10 highest m/z values, and these values were subjected to mzCloud Mass Spectral Database for compound identification. The characteristic fragmentation pattern of all components was tentatively elucidated using structurally relevant product ions, and the details of compounds were identified (Tables 5, 6). Overall, 13 additional compounds were induced in the 100 nM BRD4770-treated culture, of which 6 compounds were identified in positive (Figure 4B and Table 5) and 7 in negative (Figure 4D and Table 6) ionization mode of LC-ESI-MS/MS. A total of 14 compounds were detected in both the control and the treated cultures including 1 in positive and 13 in negative mode, respectively (Figure 4 and Supplementary Table 2). However, two compounds, i.e., nimesulide (Supplementary Figure 5A) and N-(2-oxo-3-azepanyl)benzenesulfonamide (Supplementary Figure 5B) of negative mode with RT 6.89 and 11.90, respectively, were not identified in the treated crude extract as compared to the control.

**TABLE 5:** One hundred nanomolar BRD4700-treated induced compound in the positive ionization mode.

**RT**	**M.W**	**Query spectra fragmentation (m/z+)**	**Library fragmentation spectra (m/z+)**	**Compound name**	**Chemical formula**	**Fragment similarity %**	**Function**	**References**
1.18	180.06	MS1→181→110.1	MS1→110.07	Theobromine	C_7_H_8_N_4_O_2_	74.5	Antioxidant	Azam et al., 2003
2.54	187.06	MS2→115.1	MS1→143.68 MS2→115.05	Indole-3-acrylic acid	C_11_H_9_NO_2_	83.7	Antibacterial	Cadelis et al., 2021
4.59	354.24	MS2→227.2	MS1→317.21 MS3→273.22 MS4→255.21 MS5→227.18	Carnosine	C_9_H_14_N_4_O_3_	77.3	–	–
5.46	320.01	MS2→ 320.9→303	MS1→321.01	Lorazepam	C_15_H_10_Cl_2_N_2_O_2_	79.5	–	–
10.49	194.08	MS2→195.1→149.1	MS2→195.08	Caffeine	C_8_H_10_N_4_O_2_	97.8	Antioxidant	Azam et al., 2003
12.36	193.07	MS2→194.0→ 105.1	MS1→194.08 MS2→105.03	Methylhippuric acid	C_10_H_11_NO_3_	80.7	Cytotoxic activities	Moreno et al., 2016

*–, not mentioned.*

**TABLE 6:** One hundred nanomolar BRD4700-treated induced compound in the negative ionization mode.

**RT**	**M.W**	**Query fragmentation spectra (m/z-)**	**Library fragmentation spectra (m/z-)**	**Compound name**	**Chemical formula**	**Fragment similarity%**	**Function**	**References**
1.19	155.99	MS2→154.9→111.0	MS1→111.0	2-Chlorobenzoic acid	C_7_H_5_ClO_2_	77.6	–	–
3.96	264.03	MS2→245.01	MS1→245.01 MS3→165.05 MS4→150.03 MS5→121.02	4-Hydroxy-3-methoxyphenylglycol sulfate	C_9_H_12_O_4_	83.4	–	–
7.37	162.03	MS2→117.0	MS1→161.02 MS2→117.03	4-Hydroxycoumarin	C_9_H_6_O_3_	86.4	Anticoagulants	Bye and King, 1970
9	304.24	MS2→303.1	MS1→303.23	CP 47,497-C6-Homolog-	C_20_H_32_O_2_	80.9	–	–
10.20	306.25	MS2→305.2→267.2	MS1→305.24 MS2→289.22 MS3→267.21	8Z,11Z,14Z-Eicosatrienoic acid		91.4	–	–
13.40	336.36	MS2→336.1→292.1 →249.1	MS1→336.12 MS2→292.09 MS3→264.10 MS4→249.07	Berberine	C_20_H_18_NO_4_	97	Antimicrobial	Chu et al., 2016
19.1	206.16	MS2→205.4	MS1→205.15	2,6-di-tert-Butylphenol	2,6(CH_3_)_3_C)_2_C_6_H_3_OH	97.3	Antioxidants	Papanastasiou et al., 2006

*–, not mentioned.*

### HPLC-ESI-MS/MS Identification of Induced Compounds (Positive Ion Mode) in Treated Culture

Theobromine—It showed loss of isocyanic acid (O–C = NH) [M+H–43]^+^, produced ions at m/z 138.1, further loss of a CO unit (28 Da), and produced ions at m/z 110 (Supplementary Figure 3A; Bianco et al., 2009). **Indoleacrylic acid**—Major m/z 188.0 after removal of [M+H–72]^+^ molecular mass probably in the form of CO_2_ and CO gives rise to m/z 115.1 as a final product ion (Supplementary Figure 3B). **Carnosine**—Parent m/z 355.2 results from the loss of CO [M+H–28]^+^ gives 227.2 as a product ion (Supplementary Figure 3C). **Lorazepam**—Probably by loss of water [M+H–18]^+^ forms a stable aromatic structure (Supplementary Figure 3D). **Caffeine**—The protonated molecule of caffeine at m/z 195 gives a major product ion. The ion at m/z 149.1 is the result of a ring contraction with loss of acetaldehyde (CH_3_CH_2_OH) [M+H–44]^+^ (Supplementary Figure 3E; Bianco et al., 2009). **Methylhippuric acid**—Parent ion m/z 194.0814 after loss of NHCH_2_COOH [M+H–74]^+^ and further loss of CH_3_ [M+H–15]^+^ gives product m/z 105 (Supplementary Figure 3F; Ohashi et al., 2006). Induced compounds of this positive ion mode are enlisted (Table 5).

### HPLC-ESI-MS/MS Identification of Induced Compounds (Negative Ion Mode) in Treated Culture

**2-Chlorobenzoic acid**—Deprotonated molecule gives m/z 154.9 a major product ion. The ion at m/z 111.0 is the result of loss of CH_3_CHO [M–H–18] (Supplementary Figure 4A). **4-Hydroxy-3-methoxyphenylglycol sulfate**—Parent (m/z) 263.0 resulted to m/z 245 by removal of H_2_O [M–H–18]^–^ (Supplementary Figure 4B). **Hydroxycoumarin**—Parent m/z 161 gives m/z 117 product ion after removal of CH_3_CHO [M–H–44] moieties (Supplementary Figure 4C). **CP47, 497-C6-Homolog** was found as an m/z 303.1 major product ion (Supplementary Figure 4D). **8Z, 11Z, 14Z-Eicosatrienoic acid**—Parent m/z 305.2 after removal of [M–H–38]^–^ gives 267.2 as a product ion (Supplementary Figure 4E). **Berberine**—Parent (m/z) 336.1 gives m/z 292.1 ion presumably resulting from loss of CH_3_CHO [M–H–44]^–^ and further m/z 249.1 resulted from loss of COCH_3_ [M–H–43]^–^ moieties (Wang et al., 2017; Supplementary Figure 4F). **2,6-di-tert-Butylphenol** gives m/z 205.4 as a final product of negative ion (Supplementary Figure 4G). Induced compounds of this negative ion mode are also mentioned (Table 6).

### RP-HPLC Analysis Result of Major Components, Berberine, Caffeine, and Theobromine

**FIGURE 5:**
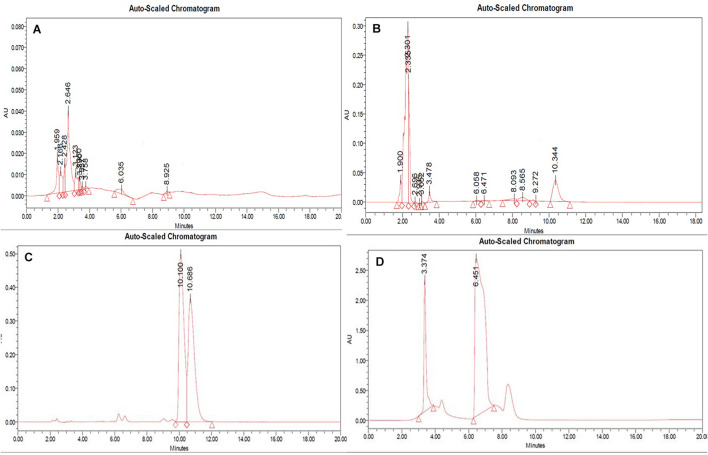
RP-HPLC **(A)** non-treated, **(B)** 100 nM BRD4770-treated crude metabolite of *D. longicolla*, **(C)** berberine standard, and **(D)** caffeine + theobromine standard.

**FIGURE 6:**
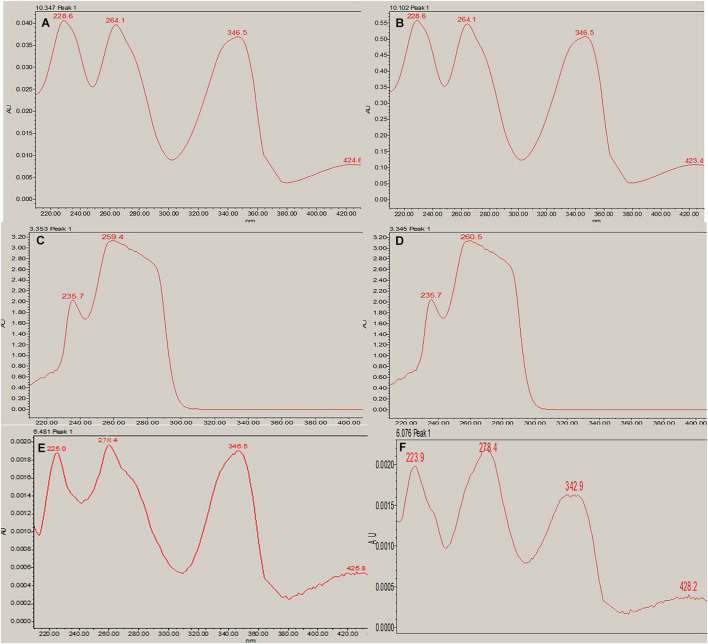
RP-HPLC absorption maxima. **(A)** Berberine standard at a given wavelength of 346.5 nm (RT-10.347). **(B)** Berberine in the 100 nM BRD4770-treated crude metabolite at a given wavelength of 346.5 nm (RT-10.102). **(C)** Caffeine standard at a given wavelength of 259.4 nm (RT-3.353). **(D)** Caffeine in 100 nM BRD4770-treated crude metabolite at a given wavelength of 260.5 nm (RT-3.345). **(E)** Theobromine standard at a given wavelength of 278.4 nm (RT-6.451). **(F)** Theobromine in 100 nM BRD4770-treated crude metabolite at a given wavelength of 278.4 nm (RT-6.076).

In RP-HPLC, the RT of the standard solution of berberine (RT-10.100) (Figure 5C), caffeine (RT-3.374), and theobromine (RT-6.451) (Figure 5D) was found, showing absorption maxima at 346.5 nm (Figure 6A; Chan et al., 2007), 259.4 nm (Vu et al., 2020; Figure 6C), and 278.4 nm (Caudle et al., 2001; Figure 6E), respectively. According to several literatures, the above compounds show similar absorption spectra at a given wavelength. The RP-HPLC chromatogram of the treated crude compound of *D. longicolla* showed slight changes in RT as 10.344, 3.478, and 6.058 (Figure 5B) with similar absorption maxima at a given wavelength of 346.5 nm (Figure 6B), 259.4 nm (Figure 6D), and 278.4 nm (Figure 6F), respectively, while RP-HPLC chromatogram of non-treated crude compounds of *D. longicolla* did not show any peak of the above RT (Figure 5A).

## Discussion

Endophytic fungus *D. longicolla* is a commonly reported plant endophyte that has been isolated from a wide range of hosts and is a reliable source for a variety of bioactive compounds (Flores et al., 2013). It has been observed that in fungi, the more novel natural compounds can be obtained, and the production of already known compounds can be up-scaled through an epigenetic regulation if treated with the right epigenetic modifiers in appropriate dose (Zutz et al., 2014). Therefore, the isolate of *D. longicolla* was grown on PDB medium with different concentrations of BRD4770 to check their epigenetic effect on gene(s) responsible for the production of cryptic metabolites that are not produced under normal culturing conditions.

Epigenetic mechanisms in eukaryotes are highly dynamic phenomena that modulate gene(s) expression regulated through chromatin structure, i.e., mainly controlled by DNA methylation, acetylation, phosphorylation, non-coding RNAs, nucleosome remodeling histone variants, and post-translational modifications (PTMs) of histone. The “histone code” consists of specific patterns of PTMs (post-translational modification) with flexible and charged N-terminus amino acids rich in arginine and lysine residues on histone tails that regulate chromatin compaction and nucleosome positioning in facultative heterochromatin (Lachner et al., 2003). Enzymes (proteins) that have a direct involvement in PTMs of histones include histone-lysine N-methyltransferases (PKMTs) and histone-arginine N-methyltransferases (PRMTs), which catalyze the transfer of one, two, or three methyl groups to lysine and arginine residues of histone proteins. Di- and tri-methylation of lysine 9 on histone H3 (H3K9Me2 and H3K9Me3) mediated by PKMT typically associated with repression (Vastenhouw and Schier, 2012). BRD4770 acts as an inhibitor to H3K9Me2, H3K9Me3, and PKMT. Methylation of H3K9 in humans is controlled by G9a [euchromatic histone-lysine N-methyltransferase 2 (EHMT2)] (Bannister et al., 2001). BRD4770 inhibited G9a with an IC_50_ of 6.3 μM and was selective for G9a, DNMT (DNA methyl transferase), and HDAC (histone deacetylase) (Kubicek et al., 2007).

In the present study, it was noticed that the crude extract of *D. longicolla* culture treated with 100 nM BRD4770 performed better in antioxidant and antibacterial assays. The EC_50_ value of the treated crude extract was much higher compared to that of the non-treated control. Moreover, the antibacterial activity was also increased against MRSA and *S. aureus* by the respective concentration of the BRD4770-treated crude extract. The findings suggest that the presence of particular concentration of BRD4770 in the growth medium certainly stimulated the secretion of some additional active compounds into the medium, which were not produced under conditions without BRD4770. These compounds could be the reason for increased EC_50_ for antioxidant activity and enhanced antibacterial activity against tested pathogenic bacteria. It clearly indicates that some of the cryptic (silent) gene(s) or gene clusters get activated and ultimately lead to the production of cryptic metabolites. Methyl ester BRD4770 at 10 μM significantly reduced cellular levels of H3K9me2 and H3K9me3 and increased cellular levels of H3K9me1, and thus, the expression of several new genes were observed in eukaryotes (Yuan et al., 2012). In this study, different concentrations of BRD4770 (1 nM to 1 μM) were used, but only 100 nM was found to be effective for activation of cryptic metabolites in broth medium. GC-MS analysis showed the difference in the crude compound composition present in treated and non-treated cultures. One hundred nanomolar BRD4770-treated GC-MS crude chromatogram clearly indicated that the non-polar compounds (Table 3) were induced. HPLC-ESI-MS/MS analyses showed the difference in crude compound composition in treated and non-treated cultures (Tables 5, 6) that further led to confirmation of the compounds that were induced after treatment with 100 nM BRD4770. Previous reports and literature have shown that among the induced compounds identified by HPLC-ESI-MS/MS, berberine had antibacterial properties against MRSA, additionally known to enhance the inhibitory efficacy of antibiotics (Chu et al., 2016), while caffeine and theobromine have antioxidant activity (Azam et al., 2003). GC-MS and HPLC-ESI-MS/MS were used for the identification of compounds and differences in the crude chromatogram composition present in the treated and non-treated cultures, while RP-HPLC was used to find similar absorption maxima of major compounds like berberine, caffeine, and theobromine in both treated and standard chromatogram at a given wavelength in the RP-HPLC. Similar absorption maxima strongly confirm the presence of these compounds. Induction of berberine may be the reason behind an increased antibacterial activity against *MRSA*. The antibacterial activity of the crude compound was found only against MRSA and *S. aureus* while *E. coli, S. boydii*, and *K. pneumoniae* were unaffected, probably because of resistance genes present within plasmids of the unaffected bacteria (Radstrom et al., 1991). The treated crude extract had shown increased antioxidant characteristics, which could be justified by the presence of caffeine and theobromine, which certainly were induced due to BRD4770 treatment. Based on the results obtained from this study and the mode of action of BRD4770 mentioned in literature, it is assumed that 100 nM BRD4770, after entering fungal cells, may be involved in the modification of histone as it does inside mammalian cells. Histone modification ultimately leads to upregulation/downregulation of silent genes/or gene clusters in causing the induction and disappearance of compounds present in crude extract. It would be contextual to mention that the additional compounds observed in GC-MS, HPLC-ESI-MS/MS, and RP-HPLC analyses may not represent completely all the additional or novel compounds induced in treated fungus, but it certainly enhances our understanding about the effects of the epigenetic modulator BRD4770 on a microbial system, the endophyte *D. longicolla*.

## Conclusion

The crude metabolite of the endophytic fungus *D. longicolla* was found to have potent antioxidant and antibacterial activity, which were selected for the treatment of the epigenetic modulator BRD4770. The dose of 100 nM BRD4770 used to treat cultures of the endophytic fungus *D. longicolla* was noted as an effective concentration in inducing the isolation of bioactive cryptic metabolites, thereby increasing antibacterial and antioxidant activities. A comparative study of BRD4770-treated and non-treated crude chromatograms of RP-HPLC with standard solutions of berberine, caffeine, and theobromine confirms the presence of respective compounds in treated cultures. This study successfully establishes the importance of BRD4770, which also interacts with epigenetic targets and can significantly induce and downregulate the production of cryptic metabolites in the endophytic fungus *D. longicolla*. The histone level modification brought out by the treatment of BRD4770 will further help to explore the exact mechanisms/pathways involved.

## Data Availability Statement

The datasets presented in this study can be found in online repositories. The names of the repository/repositories and accession number(s) can be found in the article/Supplementary Material.

## Author Contributions

JN, RK, and AS hypothesized the research work, wrote the manuscript, analyzed the data, and acquired funding. JN, PP, and RB performed the research. VS, AS, and VG analyzed the data. RK reviewed the manuscript and supervised the research. All authors contributed to the article and approved the submitted version.

## Conflict of Interest

The authors declare that the research was conducted in the absence of any commercial or financial relationships that could be construed as a potential conflict of interest.

## Publisher’s Note

All claims expressed in this article are solely those of the authors and do not necessarily represent those of their affiliated organizations, or those of the publisher, the editors and the reviewers. Any product that may be evaluated in this article, or claim that may be made by its manufacturer, is not guaranteed or endorsed by the publisher.
