# Supplementary material for: Effect of the Histone Methyltransferase Specific Probe BRD4770 on Metabolic Profiling of the Endophytic Fungus Diaporthe longicolla
Source: Front Microbiol. 2021 Sep 17;12:725463. doi: 10.3389/fmicb.2021.725463 (PMC8513106; doi:10.3389/fmicb.2021.725463)
Supplement: Supplementary file 1 [file Data_Sheet_1.docx]

**
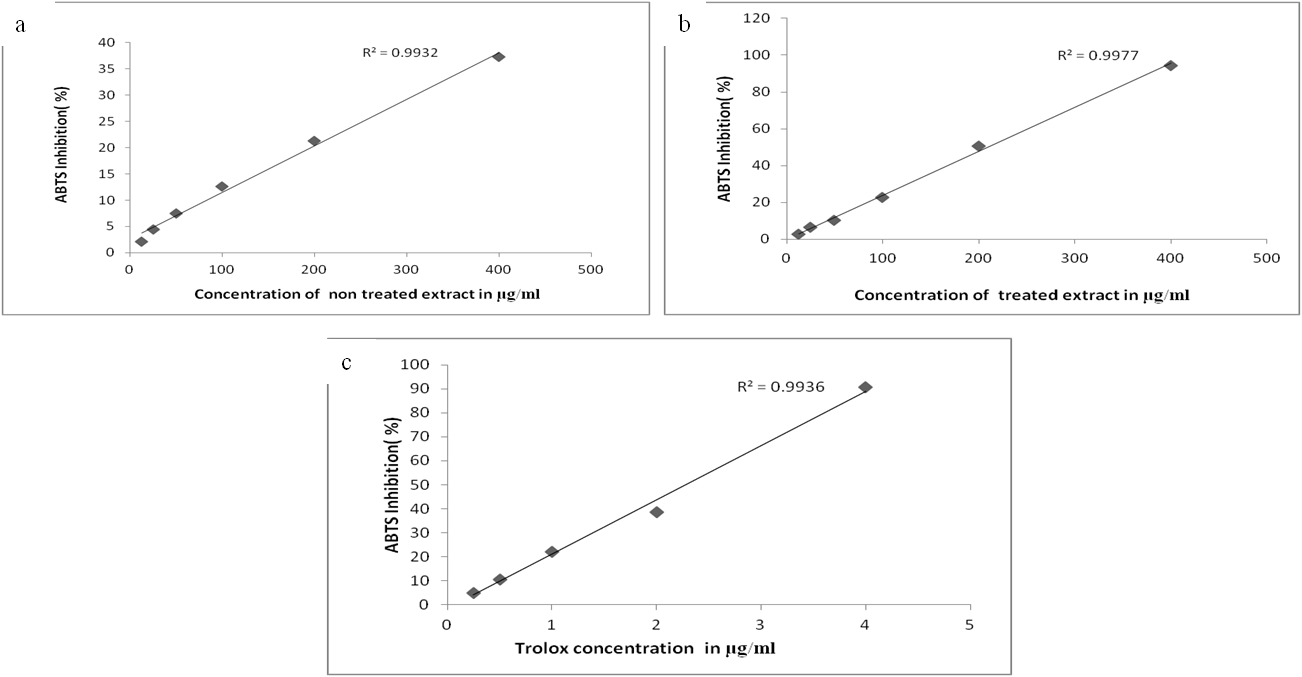
**

**Figure 1|** ABTS inhibition of non treated and treated fungal crude extract of *D. longicola* (**a**) ABTS % inhibition of non treated crude. (**b**) ABTS % inhibition of treated crude. (**c**) ABTS inhibition of trolox as a reference

**
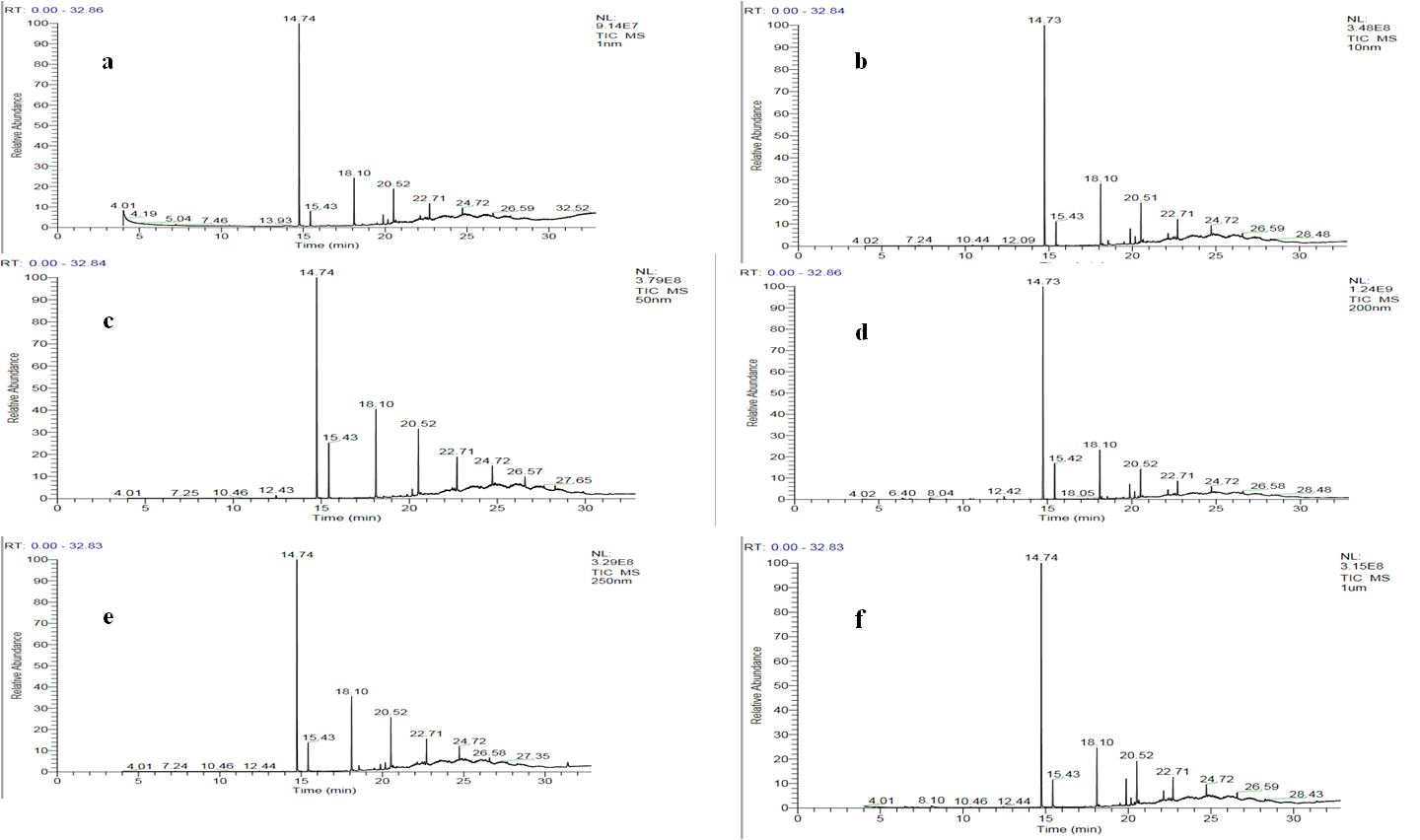
**

**Fig. 2|** GC-MS chromatogram of crude metabolites of *D. longicolla* cultures treated with BRD4770 (**a**) 1nm (**b**) 10nm (**c**) 50nm (**d**) 200nm (**e**) 250nm and (**f**) 1000nm


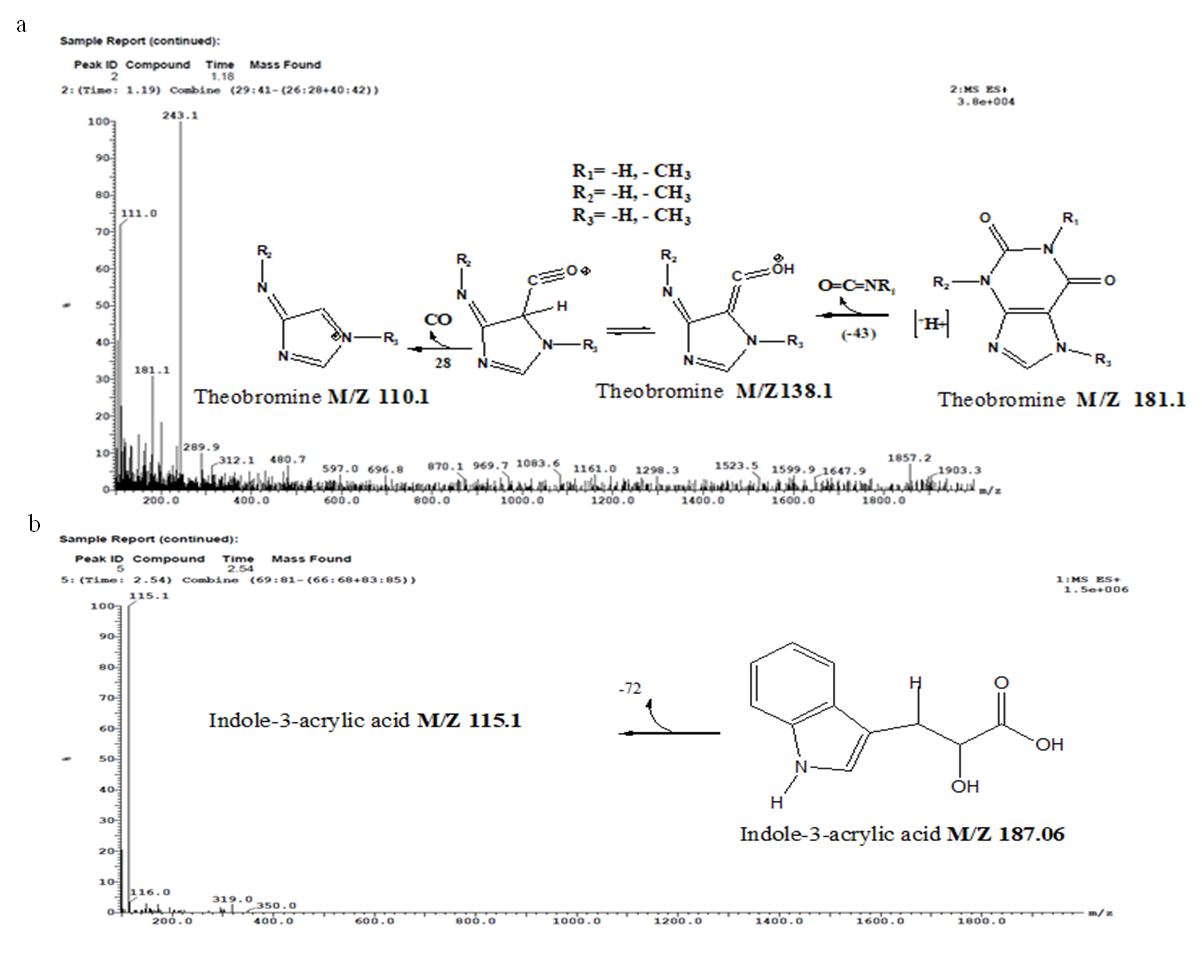


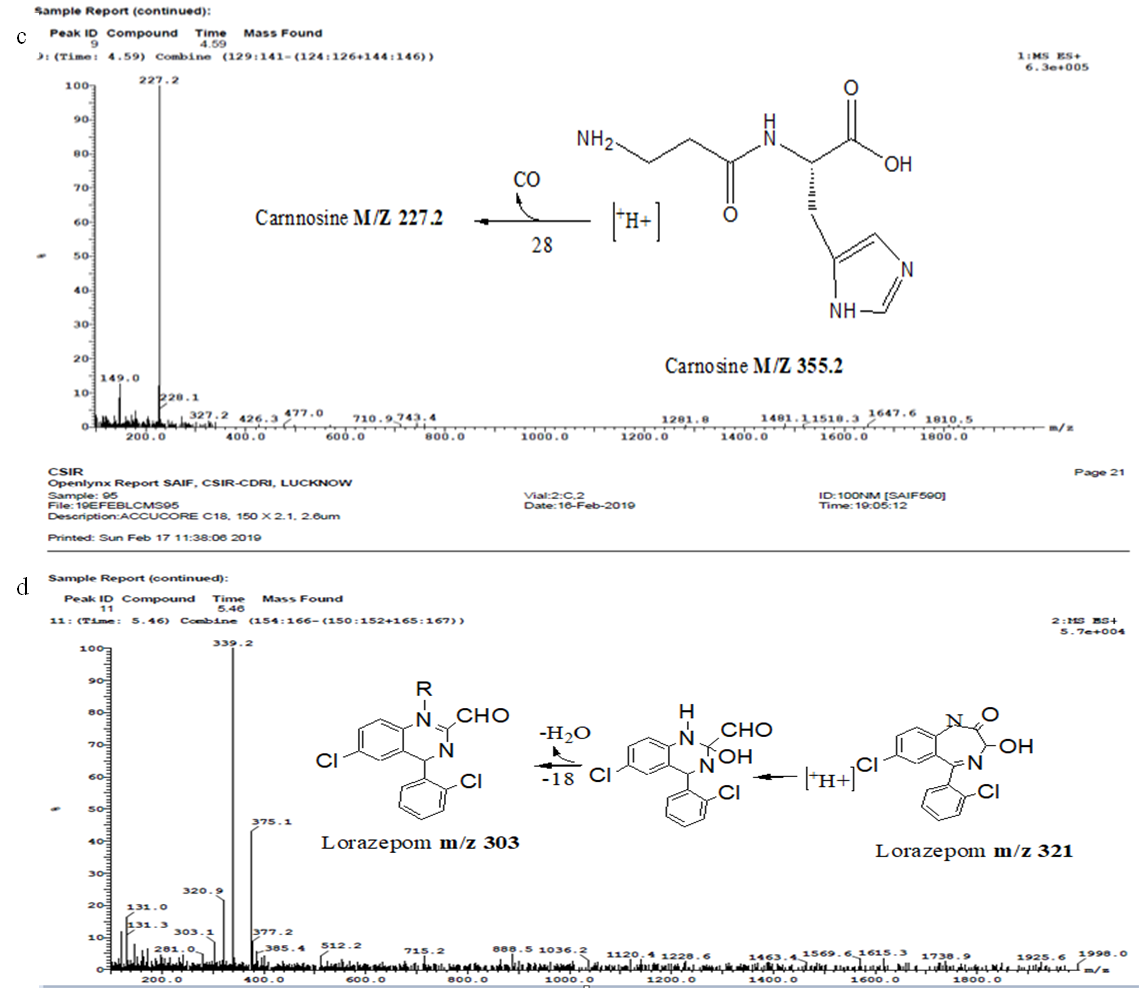


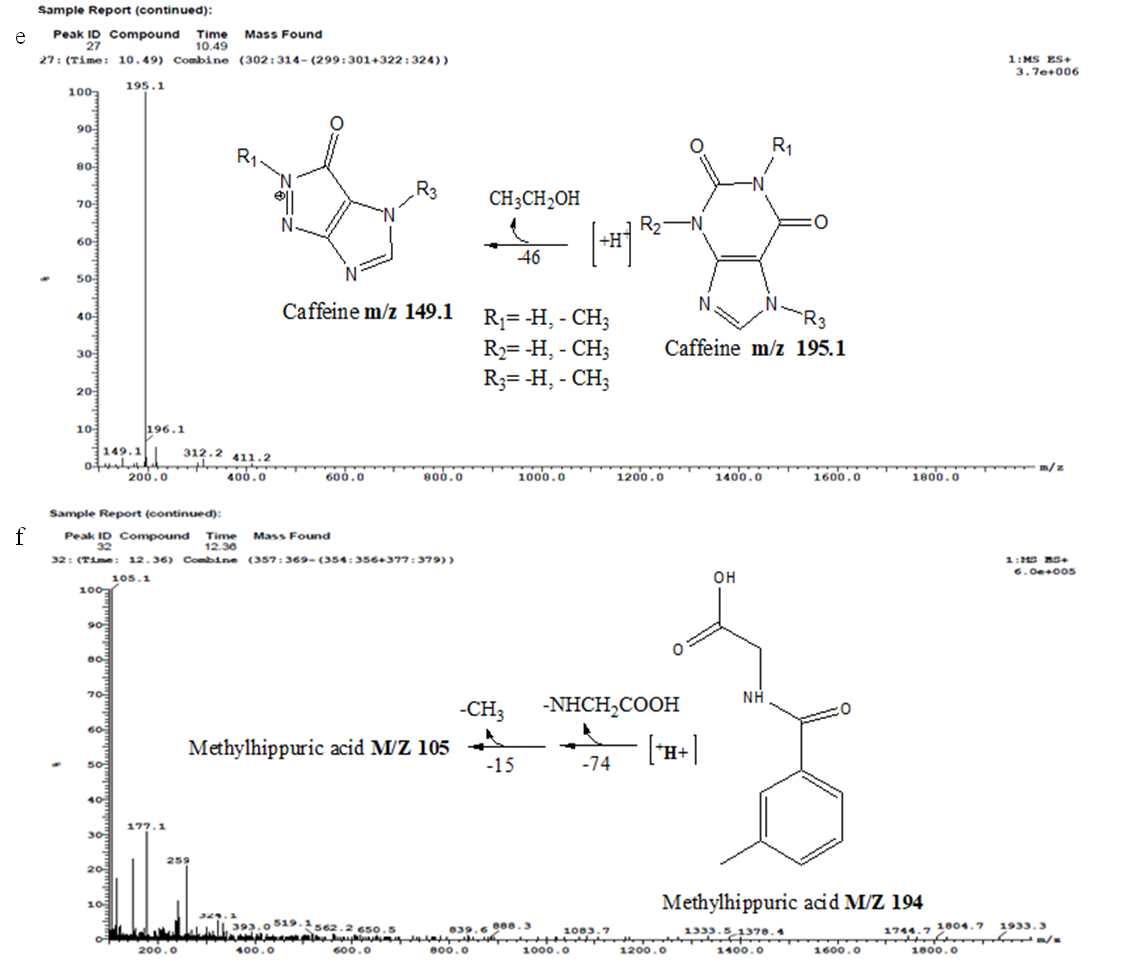


**Fig 3|** Proposed fragmentation scheme and fragmentation chromatogram of the protonated molecules in positive ESI mode (**a**)Theobromine (**b**) Indol 3 acrylic acid (**c**) Carnosine (**d**) Lorazepom (**e**)Caffeine (**f**) Methyl hippuric acid in positive ESI mode.


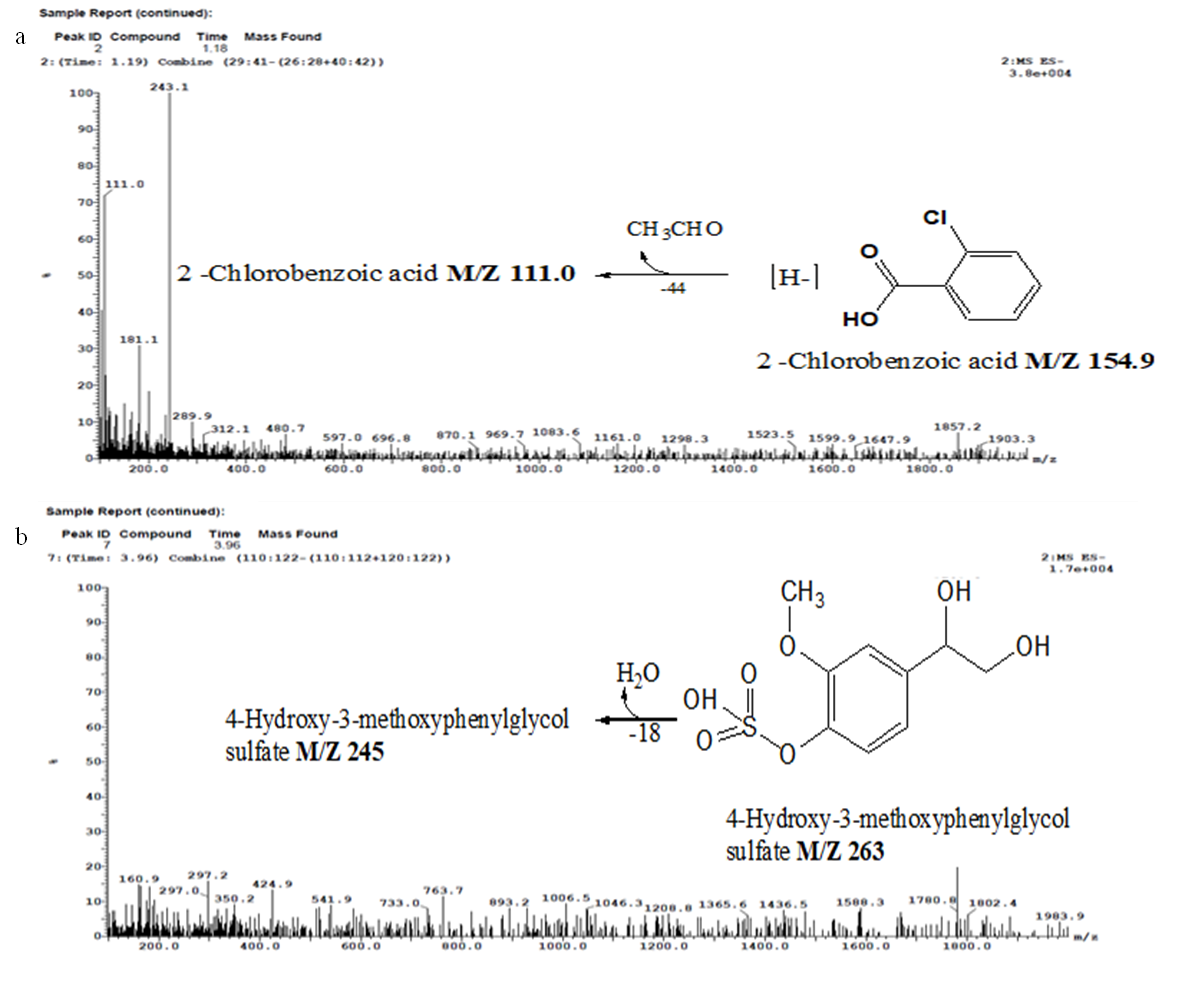


**
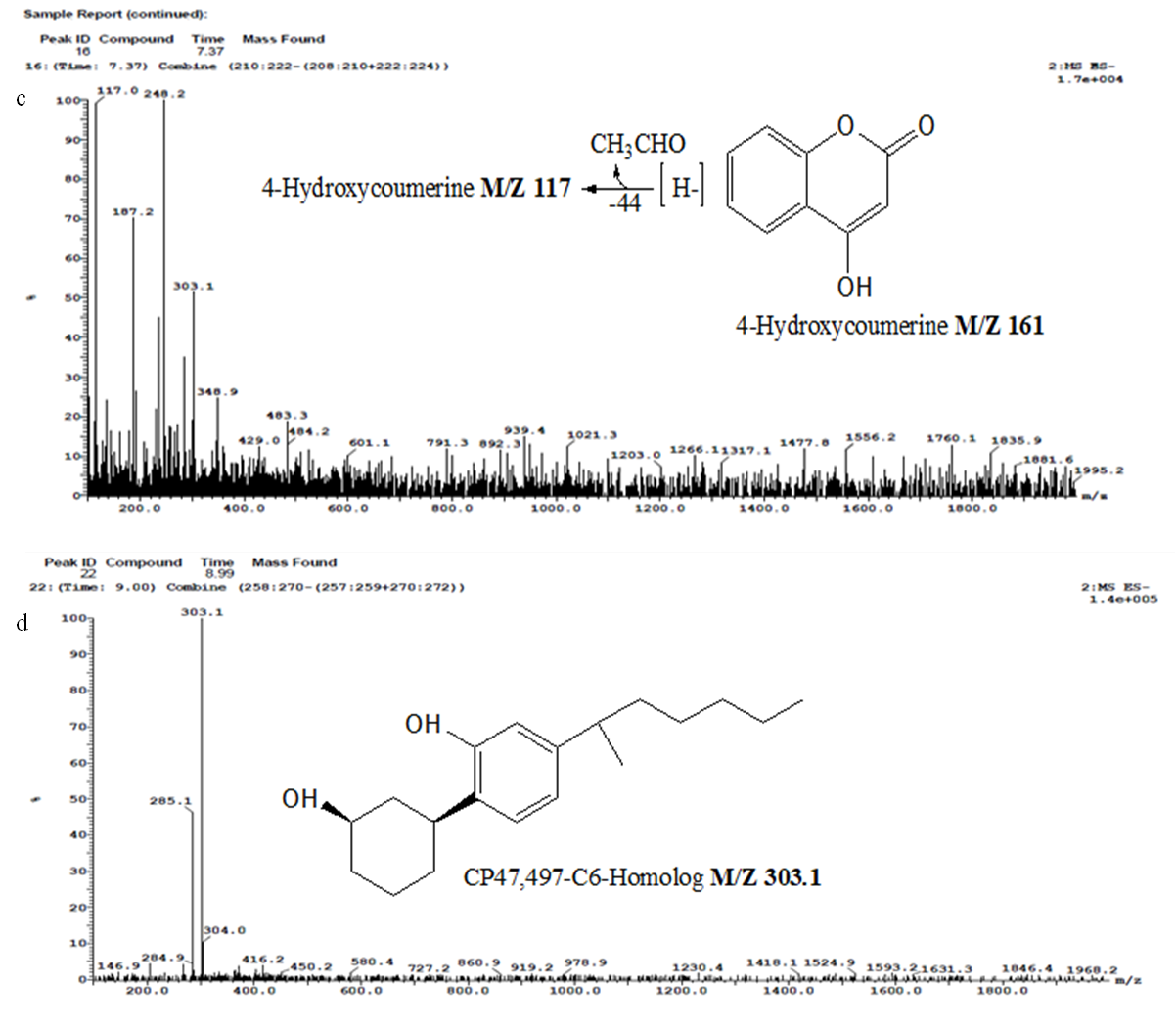
**

**
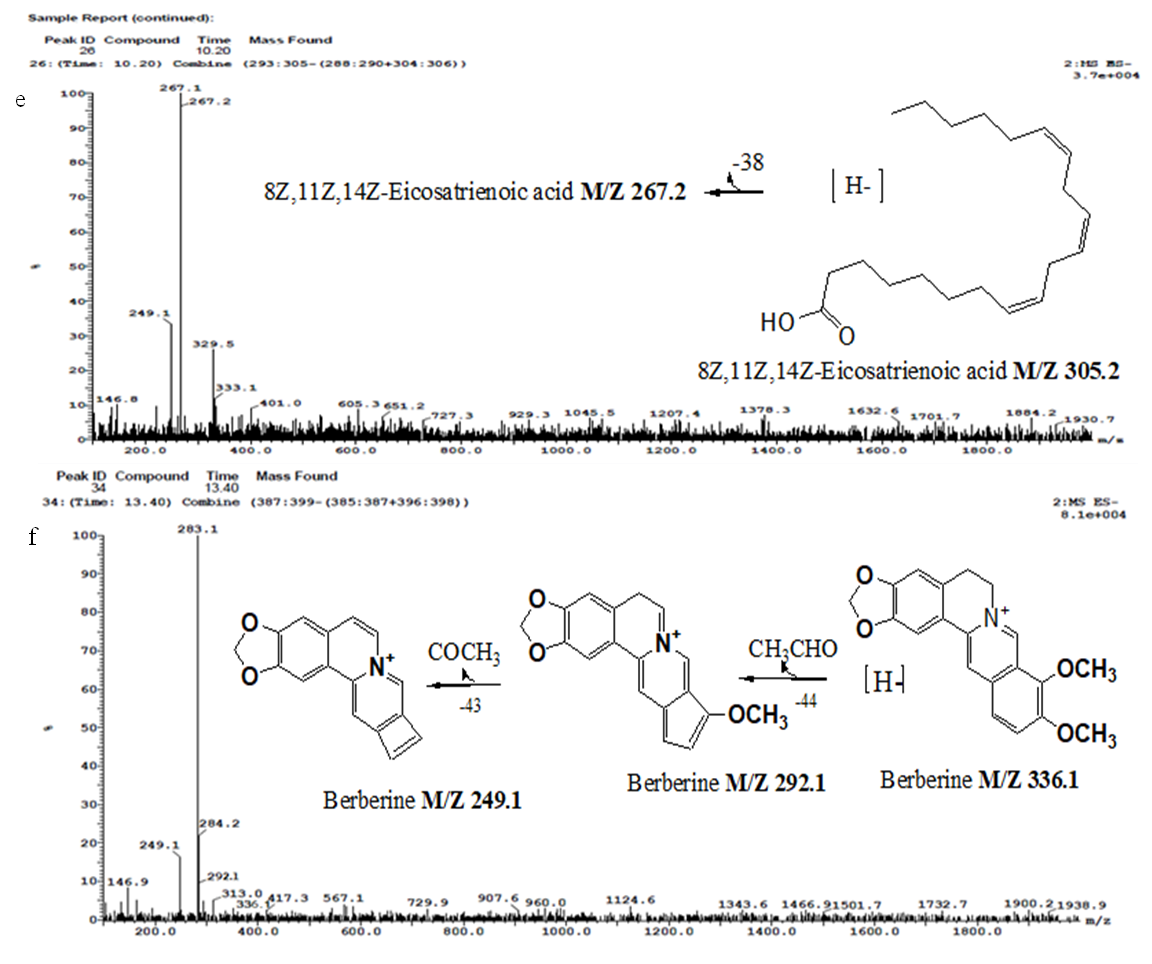
**

**
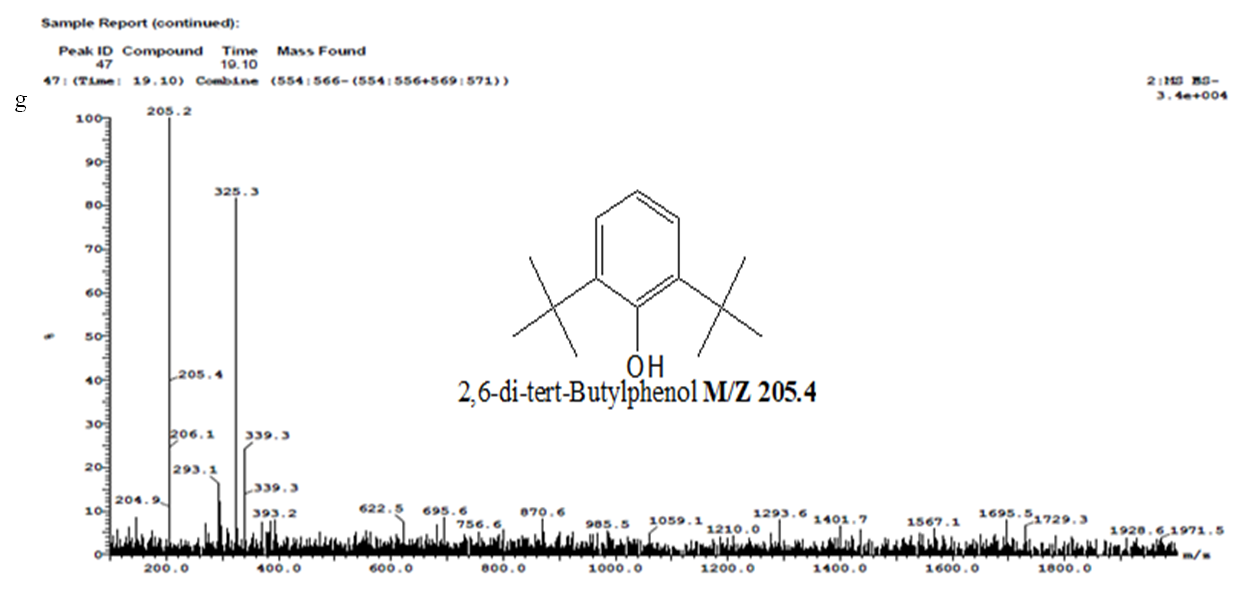
**

**Fig 4|** Proposed fragmentation scheme and fragmentation chromatogram of the deprotonated molecules in negative ESI mode (**a**)2-Chlorobenzoic acid (**b**) 4-Hydroxy-3-methoxyphenylglycol sulfate (**c**) 4-Hydroxycoumarin (**d**) CP47,497-C6-Homolog (**e**) 8Z,11Z,14Z-Eicosatrienoic acid (**f**) Berberine (**g**) 2,6-di-tert-Butylphenol in positive ESI mode

**
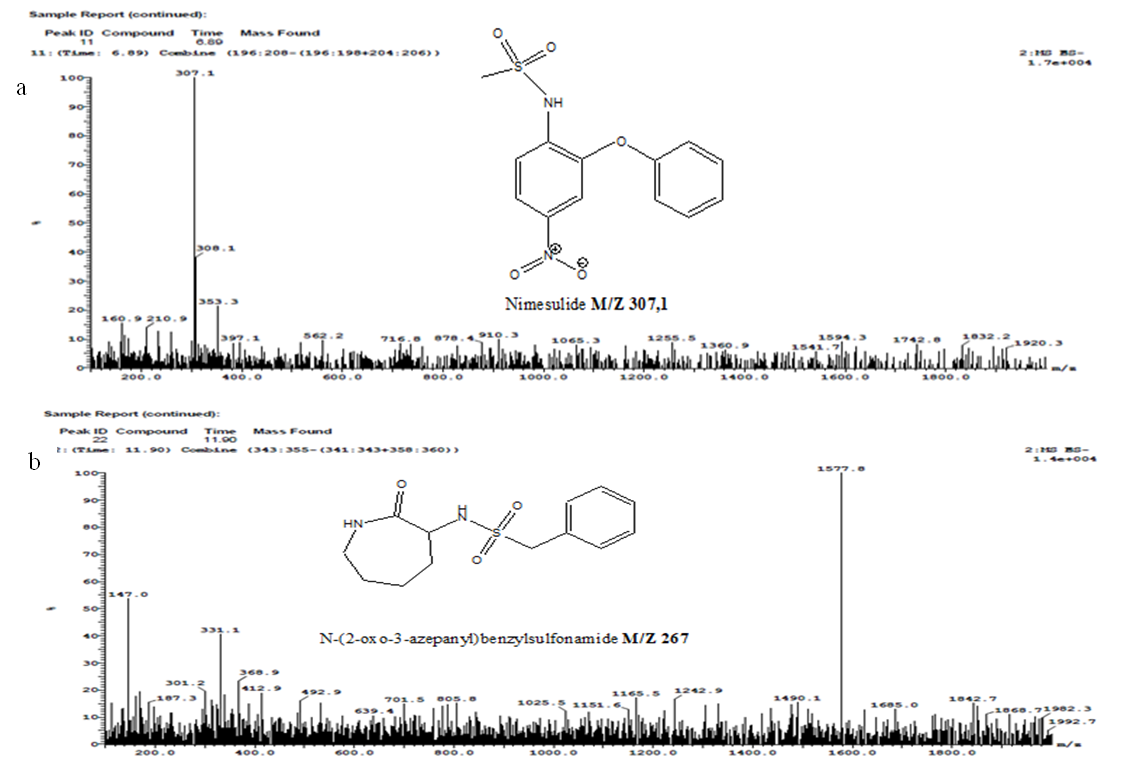
Fig 5**| Fragmentation chromatogram of missing compound (**a**) Nimesulide with RT 6.89 (**b**) N-(2-oxo-3-azepanyl)benzylsulfonamide with RT 11.90 after treatment with 100nm BRD4770

**Table1|**These are the compound which was found in the both treated and untreated culture of the positive ionization mode

| **RT-Control metabolite** | **RT-100nm BRD4700 treated metabolite** | **Mass Matches(m/z+)** | **Name of compound** |
| --- | --- | --- | --- |
| 18.64 | 18.88 | 301.2→205.1→1149 | Oleic acid |

**Table2|** These are the compound which was found in the both treated and untreated culture with some slight modification in the RT and some was found as the same (negative ionization mode).

| **RT-Control metabolite** | **RT-100nm**  **treated**  **metabolite** | **Mass Matches of both 100nm BRD4770 treated and non treated crude metabolite (m/z-)** | **Name of compounds** |
| --- | --- | --- | --- |
| 1.5 | 1.5 | 117.1 | Cinnoline-3,4-diol |
| 1.88 | 1.91 | 136.04 | Salicylamide |
| 5.46 | 5.46 | 375→339.2→303 | Lorazepom |
| 7.06 | 7.06 | 308.1→ 307.1 | Phloretin |
| 7.91 | 7.91 | 267.2→ 207.1 | 8Z,11Z,14Z-Eicosatrienoic acid |
| 8.32 | 8.32 | 285.1→ 284.7 | 8Z,11Z,14Z-Eicosatrienoic acid |
| 10.81 | 10.78 | 386→269.2→ 267 | Epigallocatechin |
| 13.64 | 13.61 | 283.1→ 194 | Difenoxurone |
| 14.36 | 14.36 | 149.60 | Hydrocinnamic acid |
| 17.56 | 17.50 | 621→327→326→ 325.2 | N-(1-adamantyl)-N^,^-(2,2-diethoxyethyl)thiourea |
| 18.79 | 18.88 | 325.2 | Hydrocinnamic acid |
| 19.99 | 20.02 | 339.2→311.2→ 299 | Arachidonic acid |
| 24.59 | 24.59 | 339.4 | 4-Chloro-N-{2-[(5-phenylthieno[2,3-d]pyrimidin-4-yl)amino]ethyl}benzenesulfonamide |

**Highlights**

- An endophytic fungus (L3), isolated from the leaf tissues of *Saraca asoca* was identified as *Diaporthe longicolla*.
- The crude metabolite extracts of *D. longicolla* showed strong antioxidant and antibacterial properties.
- *D*. *longicolla* was treated with different concentrations of BRD4770 for evaluation of its antibacterial and antioxidant potential
- The crude extract obtained from cultures treated with 100nM concentration of BRD4770, showed increased inhibition zone against *S. aureus* and methicillin-resistant *Staphylococcus aureus* (MRSA), compared to the non-treated control*.*
- Antioxidant activity was also founded to increase in treated with 100nM BRD4770 compared to the non-treated culture
- The composition of non-treated as well as treated crude extract was analyzed and induced compounds were identified with the help of GC-MS and LC-ESI-MS/MS.
- LC-ESI-MS/MS analysis showed berberine (antibacterial), caffeine and theobromine (antioxidant) like compounds etc. were induced in BRD4770 treated crude extract
- Presence of particular absorbance at wavelength 346.5 for berberine, 259.4 for caffeine and 278.4nm for theobromine in the reverse phase HPLC analysis of both BRD4770 treated crude metabolites and standard solution confirms its presence.
